# Rift Valley fever virus infects trophoblast cell lines in an LRP1-dependent manner

**DOI:** 10.1099/jgv.0.002300

**Published:** 2026-08-12

**Authors:** Rachael E. Rush, David A. Price, Cynthia M. McMillen, David L. Donermeyer, Daisy W. Leung, Gaya K. Amarasinghe, Amy L. Hartman

**Affiliations:** 1 Center for Vaccine Research, School of Medicine, University of Pittsburgh, Pittsburgh, PA, USA; 2 Department of Infectious Diseases and Microbiology, School of Public Health, University of Pittsburgh, Pittsburgh, PA, USA; 3 Department of Medicine, Washington University School of Medicine in St. Louis, St. Louis, MO, USA; 4 Department of Pathology and Immunology, Washington University School of Medicine in St. Louis, St. Louis, MO, USA

**Keywords:** bunyavirus, CD91, LRP1, placenta, Rift Valley fever, trophoblast

## Abstract

Rift Valley fever virus (RVFV) is a mosquito-borne phlebovirus that poses a growing global threat, causing severe disease in humans and livestock, including hepatitis, haemorrhage and encephalitis. Documented cases of vertical transmission in humans and livestock highlight risks during pregnancy. Although RVFV can directly infect the placenta, how it enters trophoblast cells remains unclear. Here, we investigated the role of low-density lipoprotein receptor-related protein 1 (LRP1), a known RVFV entry factor, in human trophoblast infection. Using JEG-3, JAR and HTR-8/SVneo trophoblast cell lines, we demonstrate that RVFV replicates to high titres and LRP1 expression varies across cell types. Competitive inhibition assays using the high-affinity LRP1 ligand murine RAP domain 3 and recombinant LRP1 fragments (cluster proteins) indicate that RVFV requires LRP1 for efficient trophoblast infection. Together, these findings implicate LRP1 as a determinant of RVFV infection at the maternal-fetal interface.

## Introduction and Results

Members of the low-density lipoprotein (LDL) receptor (LDLR) family are an evolutionarily conserved group of cell surface proteins that have recently been identified to mediate cellular entry of a variety of emerging vector-transmitted viruses [1–6]. One such receptor, low-density lipoprotein receptor-related protein 1 (LRP1), is a host entry factor for Rift Valley fever virus (RVFV) and the peribunyaviruses Oropouche and Jamestown Canyon viruses (OROV and JCV, respectively) [2, 4, 6]. LRP1 is expressed on a variety of cells and tissues that are permissive to RVFV infection, including prominent expression in placental tissue [7]. Notable outcomes of RVFV infection in ruminants include waves of fetal loss in pregnant livestock [8–10]. Alarmingly, vertical transmission has been documented in a small number of human cases [11–15], and women infected with RVFV are up to four times more likely to have a second or third trimester miscarriage [15]. Furthermore, several cases of in utero infection and microcephaly were reported in a surge of OROV cases in South America in 2024 [16–18]. Given that both RVFV and OROV are suspected to cause in utero infection, and both rely on LRP1 for efficient cellular infection, we hypothesized that RVFV may take advantage of LRP1 to infect placenta cells.

Differentiated trophoblast-lineage cells play crucial roles during pregnancy: they form the outer layer of cells in the blastocyst, mediate implantation, facilitate nutrient and gas exchange, produce hormones and modulate inflammation [19–21]. Several immortalized human trophoblast cell lines exist and can be used to provide initial insights into cellular infection at the maternal-fetal interface. Two cell lines, JEG-3 and JAR, were derived from human choriocarcinoma epithelial cells and exhibit cytotrophoblast-like morphology [22–24]. Both cell lines can fuse into multi-nucleated syncytiotrophoblasts without the need for chemical induction. In contrast, the HTR-8/SVneo (HTR-8) cell line exhibits extravillous morphology [25] that plays an important role in invasion and attachment of the developing fetus to the uterine wall [26]. Typically, syncytiotrophoblast cells are resistant to pathogens [20]. However, our previous work demonstrated that RVFV readily infects and replicates in primary human placenta explants, with evidence of infection of both cytotrophoblasts and multi-nucleated syncytiotrophoblasts [21]. The mechanism by which RVFV enters and infects these cells remains unclear.

**Fig. 1.**
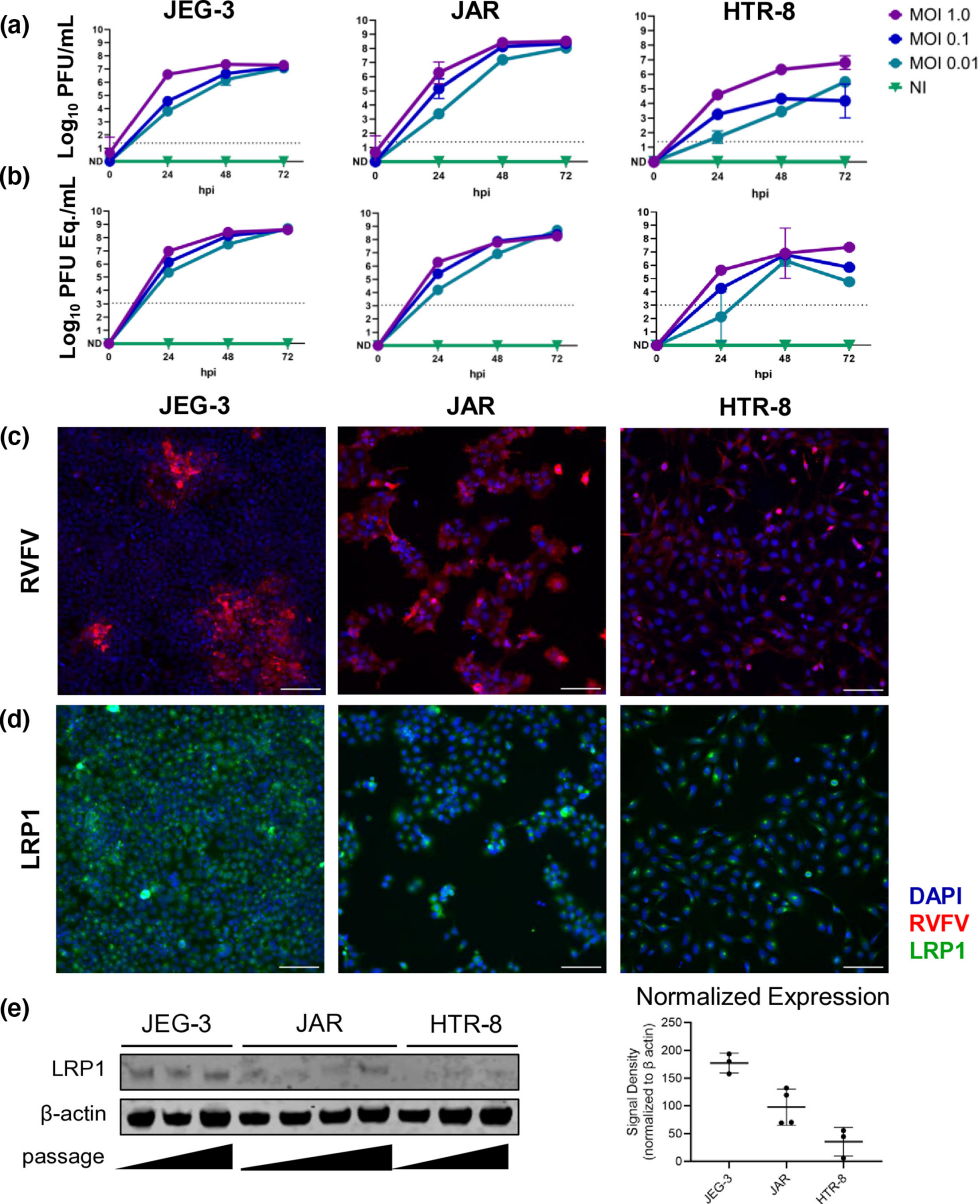
Human trophoblast cell lines support RVFV infection and express LRP1. Growth curves of RVFV ZH501 in JEG-3, JAR and HTR-8 cells at various MOIs. (**a**) Infectious titres were quantified via viral plaque assay and (**b**) vRNA was quantified via quantitative reverse transcription polymerase chain reaction (qRT-PCR) (*n*=3 replicates per dose and timepoint). Dashed lines represent the limit of detection. (**c**) Cells were stained with an anti-RVFV or separately with an (**d**) anti-LRP1 antibody. Blue=DAPI, red=RVFV nucleoprotein antigen, green=LRP1. Images obtained at 20× magnification, scale bar=100 µm. (**e**) Protein lysates were generated from JEG-3, JAR and HTR-8 cells and analysed via western blot for LRP1 and Beta-actin expression. Gel was loaded by increasing passage age (lower passage to higher passage left to right, respectively).

The baseline permissivity of JEG-3, JAR and HTR-8 cells to RVFV (virulent ZH501 strain) was measured by infection of each cell line at multiplicities of infection (MOIs) of 1.0, 0.1 and 0.01. RVFV replicated in a dose-dependent manner in all trophoblast cell lines tested, reaching infectious titres as high as 10^6^ p.f.u. (plaque-forming units) ml^−1^ in JEG-3 cells, 10^8^ p.f.u. ml^−1^ in JAR and 10^4^ p.f.u. ml^−1^ in HTR-8 cells by 48 h post-infection (hpi) at an MOI of 0.1 (Fig. 1a). RVFV viral RNA (vRNA) and infectious titres followed similar production curves (Fig. 1b), with RVFV reaching vRNA titres as high as 10^8^ p.f.u. equivalents ml^−1^ in JEG-3 and JAR cells and 10^6^ p.f.u. equivalents ml^−1^ in HTR-8 cells by 48 hpi, demonstrating that vRNA is reflective of infectious viral levels in this system. HTR-8 cells were less permissive overall to RVFV and had more variability in titres following infection. Immunofluorescent staining of RVFV nucleoprotein antigen showed more RVFV antigen detected in JEG-3 and JAR cells compared to HTR-8 at 24 hpi with an MOI 0.1 (Fig. 1c).

To examine the levels of LRP1 expression in all three cell lines, we used both immunofluorescent staining and western blot. Staining for LRP1 revealed diffuse patterns of LRP1 expression throughout JEG-3 cells and a hybrid diffuse and perinuclear staining in JAR cells, whereas HTR-8 cells displayed more perinuclear punctate staining of LRP1 (Fig. 1d). By western blot, JEG-3 cells express the most LRP1, followed by JAR and then HTR-8 cells, regardless of cellular culture passages and age (Fig. 1e). The levels of RVFV replication in each of these cells follow the same relative ranking, being the highest in JEG-3, followed by JAR and then HTR-8 cells. These data suggest that LRP1 expression levels in JEG-3, JAR and HTR-8 cells align with observed differences in RVFV infection.

**Fig. 2.**
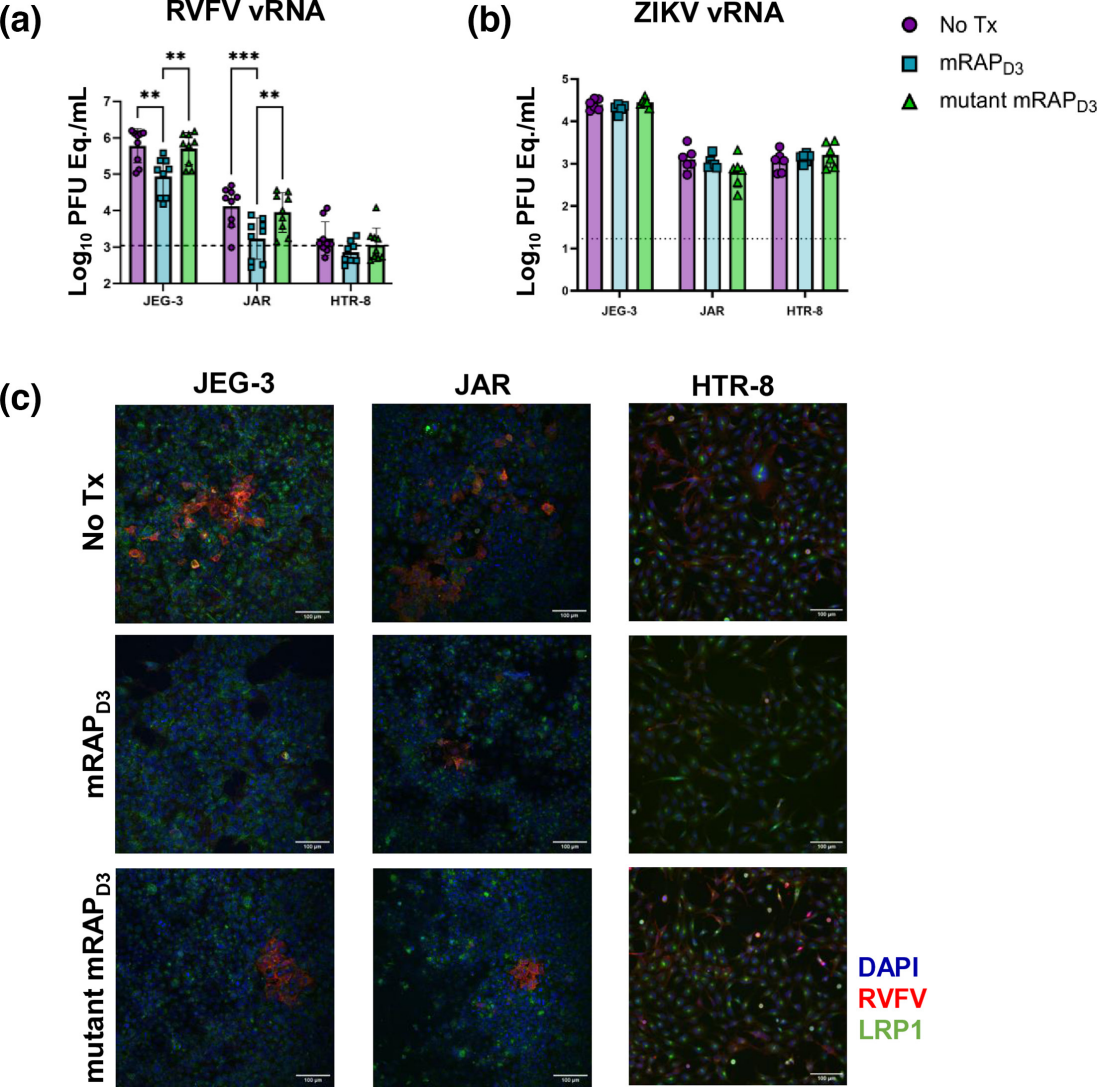
Treatment with LRP1 binding chaperone protein RAP reduces RVFV but not ZIKV titres in trophoblast cell lines. (**a**) Pre-treatment of JEG-3, JAR and HTR-8 cells with 5 µg ml^−1^ WT mRAP_D3_ prior to infection with RVFV (MOI 0.1) reduced RVFV vRNA in JEG-3, JAR and to a lesser extent HTR-8 cells compared to no treatment (no Tx). Dashed lines represent the LOD. **P<0.01, ***P<0.001. *P*-values were determined by two-way ANOVAs. vRNA data from three separate experimental replicates, each with three technical replicates. (**b**) Treatment of trophoblast cells with 5 μg ml^−1^ WT mRAP_D3_ prior to infection with ZIKV (MOI 0.1) did not have any impact on ZIKV vRNA levels measured from supernatant via qRT-PCR in JEG-3, JAR or HTR-8 cells 48 hpi. (**c**) Detectable RVFV viral antigen is reduced following 5 µg ml^−1^ mRAP pre-treatment in each of the tested cell lines 24 hpi. Images obtained at 20×. Scale bar=100 µm. Blue=DAPI, red=RVFV nucleoprotein, green=LRP1.

We employed two approaches to competitively inhibit the interaction of RVFV with LRP1. We took advantage of receptor-associated protein (RAP), a high-affinity chaperone protein that, in physiological settings, binds intracellularly to LRP1 and other LDLRs to out-compete other ligands prior to trafficking to the cell surface. RAP has highly conserved orthologs across species and binds to multiple members of the LDLR family [27, 28]. Murine RAP domain 3 (mRAP_D3_), produced recombinantly and added exogenously to cells in culture, is an effective molecular tool to block the binding of RVFV, OROV and JCV to LRP1 [2, 4, 6]. Notably, mRAP_D3_ binds to two extracellular domains of LRP1, termed clusters II and IV (CL_II_ and CL_IV_, respectively), the same regions to which RVFV glycoprotein Gn associates [2, 28, 29]. Pre-treatment of trophoblast cells with mRAP_D3_ resulted in 90% reduction of RVFV vRNA 24 hpi in JEG-3 and JAR and to a lesser extent HTR-8 cells across three independent experiments, each with three technical replicates (Fig. 2a). Relative infection levels, which represent the average titres from an experimental group (*n*=3) compared to the no-treatment control, were decreased by ∼85% each in JEG-3 and JAR cells pretreated with mRAP_D3_. However, the relative infection levels in HTR-8 cells varied widely (Fig. 2a, right panel), likely due to RVFV vRNA in HTR-8 cells being at or below the lower limit of detection (LOD) at 24 hpi (Fig. 2a, left panel). Pre-treatment of cells with a mutated version of mRAP_D3_, containing K265A/K279E mutations which abrogate binding to LRP1 [2, 4], had little impact on RVFV vRNA suggesting that the reduction in vRNA is dependent on mRAP_D3_ blocking the LRP1 binding sites. Immunofluorescence staining revealed that more RVFV antigen is detectable in JEG-3 and JAR cells that were untreated (no Tx) or treated with mutant mRAP_D3_ compared to those treated with wild-type mRAP_D3_ (Figs 2c and S1, available in the online Supplementary Material). Detection of RVFV antigen in HTR-8 cells, although low, was consistent across the treatment groups which aligns with the levels of vRNA. Since HTR-8 and similar extravillous trophoblast cells express lower levels of LRP1 [30, 31], then treatment with mRAP_D3_ is likely to have less of an impact on RVFV infection. Perhaps this also explains the relatively poor permissivity of these cells overall (Fig. 1). Furthermore, the perinuclear staining of LRP1 in HTR-8 cells may indicate an altered expression profile which may also explain the lesser impact of mRAP_D3_ on RVFV infection in these cells.

To ensure that extracellular mRAP_D3_ treatment showed specificity for inhibiting RVFV rather than a general anti-viral response unrelated to LDLR binding, we pretreated cells similarly prior to infection with Zika virus strain PRVABC59 (ZIKV; MOI 0.1), a flavivirus that is not known to interact with LRP1 or related receptors. Pre-treatment with mRAP_D3_ had no impact on ZIKV infection levels (Fig. 2b), suggesting that the reduction in RVFV vRNA and antigen staining following mRAP_D3_ treatment was specific to its function as a competitive inhibitor of LDLRs.

**Fig. 3.**
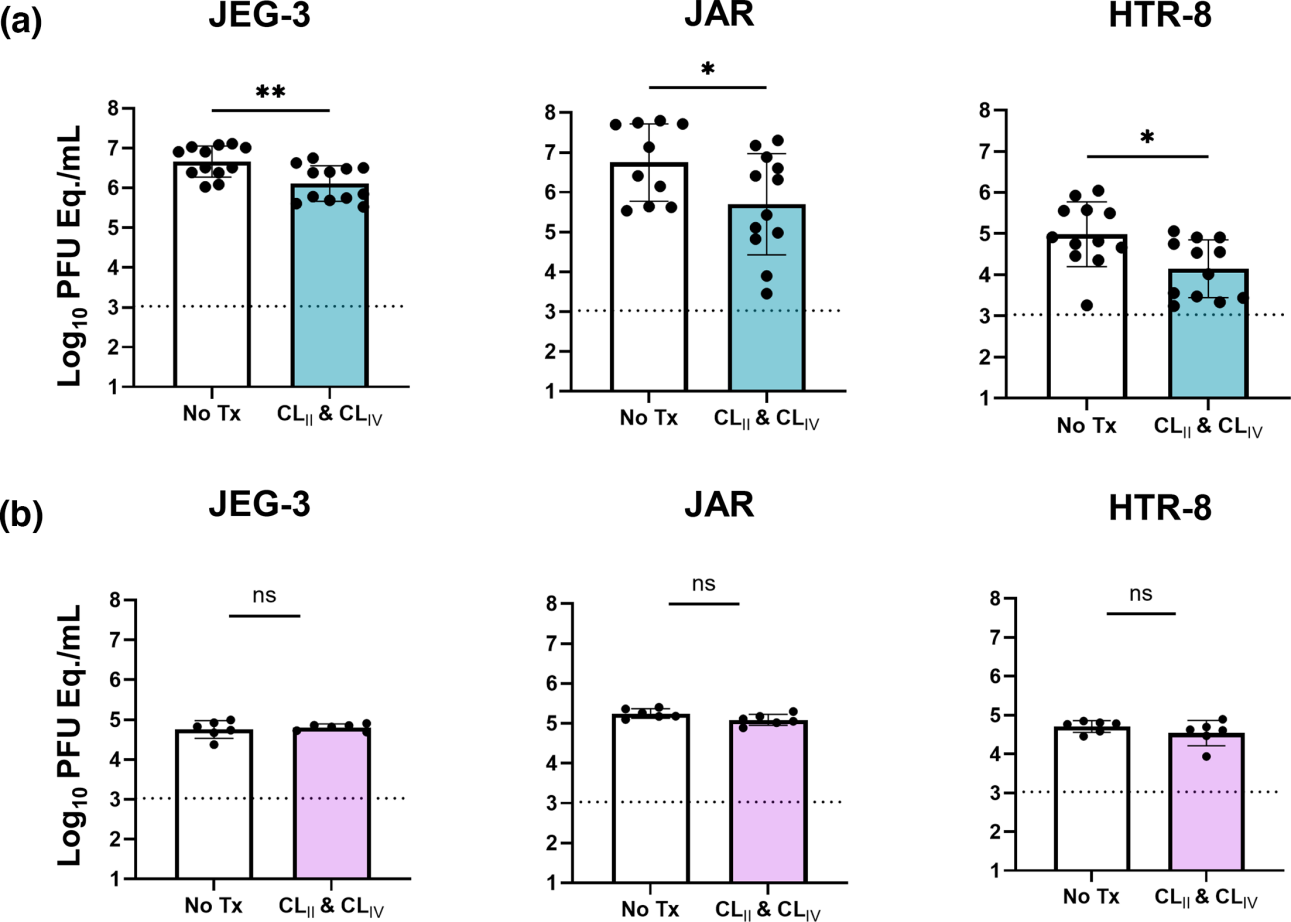
Pre-treatment with LRP1 CL_II_ and CL_IV_ reduces RVFV but not ZIKV infection in trophoblast cell lines. (**a**) Pre-treatment of RVFV ZH501 (MOI 0.1) with 5 µg ml^−1^ LRP1 CL_II_ and CL_IV_ proteins prior to infection reduced RVFV vRNA from infected JEG-3, JAR and HTR-8 cells. (**b**) Pre-treatment of ZIKV (MOI 0.1) with 5 µg ml^−1^ LRP1 CL_II_ and CL_IV_ proteins did not have any impact on ZIKV vRNA levels. Dashed lines represent the LOD. *P<0.05, **P<0.01. *P*-values were determined by unpaired two-sample t-tests, data from two (ZIKV) or three (RVFV) experimental replicates, each with at least three technical replicates per experiment, are shown.

A limitation of the data above is the fact that mRAP_D3_ binds to all LDLRs [32]. For a more specific competitive inhibitor of the RVFV-LRP1 interaction, we employed decoy receptors, consisting of LRP1 extracellular CL_II_ or CL_IV_ domains fused to human Fc, along with an untreated control. RVFV inoculum was pre-treated with a 1:1 ratio (5 μg ml^−1^) of recombinant LRP1 CL_II_ and CL_IV_ decoy proteins for 1 h prior to infection of JEG-3, JAR and HTR-8 cells as described above. Pre-treatment with decoy cluster receptors resulted in 80–90% reduction in vRNA and relative infectivity in JEG-3 and JAR and an average of 50% decrease in relative infectivity in HTR-8 cells compared to control-treated cells (Fig. 3a). CL_II_ and CL_IV_ pre-treatment had little to no impact on ZIKV vRNA levels (Fig. 3b). The lack of impact of mRAP_D3_ or LRP1 CL_II_ and CL_IV_ pre-treatment on ZIKV infection supports the finding that RVFV requires binding to CL_II_ and CL_IV_ [2], and that blocking those binding sites does not have a general anti-viral effect nonspecific to LRP1 ligand binding. These results suggest that RVFV utilizes LRP1 to bind, enter and efficiently infect JEG-3, JAR and to a lesser extent, HTR-8 cells.

Together, our findings suggest that RVFV efficiently infects and subsequently replicates in human trophoblast cell lines in an LRP1-dependent manner. As with most other cell types [2], competitive inhibition with mRAP or decoys reduces infection but does not eliminate it under the conditions tested here, leaving open the possibility of alternate receptors or entry mechanisms. The relative LRP1 expression levels and capacity for RVFV replication in JEG-3, JAR and HTR-8 cells and the inhibitory effects of LRP1-targeting reagents suggest LRP1 serves as a key entry factor for RVFV in placenta cells. Because LRP1 has highly conserved orthologs across multiple species permissive to RVFV-induced fetal infection, including sheep, cattle and goats, it is feasible that LRP1 may be an important entry factor in other species [21, 33]. Future studies are directed at determining the role of LRP1 in cross-species infection. This work provides insight into how RVFV may contribute to vertical transmission, an outcome of RVFV infection that remains underrecognized and underreported in human populations. Given the high conservation of LRP1 across mammalian species and the well-documented detrimental impacts of RVFV on ruminant pregnancies, these findings may also have broader implications for zoonotic or veterinary disease surveillance. Ongoing studies using primary placental tissue and *in vivo* models will be critical to fully elucidate the role of LRP1 in vertical transmission and explore the potential of LRP1-targeted strategies to prevent congenital RVFV infection.

## Methods

### Cell line info

JEG-3 (HTB-36), JAR (HTB-144) and HTR-8 (CRL-3271) cells were purchased from the American Type Culture Collection (ATCC). JEG-3 cells were maintained in Eagle’s Minimum Essential Medium (ATCC, 30–2003) supplemented with 10% FBS (fetal bovine sera; Sigma-Aldrich, F2442), 1% penicillin/streptomycin (Pen/Strep) and 1% l-glutamine (l-Glut). JAR cells were maintained in Roswell Park Memorial Institute 1640 medium (RPMI) (ATCC, 30–2001) supplemented with 10% FBS, 1% Pen/Strep and 1% l-Glut. HTR-8 cells were maintained in RPMI supplemented with 5% FBS, 1% Pen/Strep and 1% l-Glut. All cells were maintained in a humidified incubator at 37 °C with 5% CO_2_.

### Viral infections and biosafety

RVFV ZH501 was rescued through reverse genetics and provided by Stuart Nichol at the Centers for Disease Control and Prevention (CDC). All work with RVFV was completed in the Regional Biocontainment Laboratory (RBL) under biosafety level 3 conditions at the University of Pittsburgh. Personnel are approved under the Federal Select Agent programme to work with RVFV, and all work was overseen and approved by the appropriate biosafety committees. The PRVABC59 (Human/2015/Puerto Rico) strain of Zika virus (ZIKV) was purchased from BEI Resources (NR-50240) from the Arbovirus Reference Collection (CDC, Fort Collins, CO, USA). For infections, JEG-3, JAR and HTR-8 cells were seeded in 24-well plates at a density of 1.25E5 cells/well. The following day, the media was removed, and the viral inoculum was diluted in Dulbecco’s Modified Eagle Medium (DMEM, ATCC 30-002) supplemented with 2% FBS, 1% Pen/Strep and 1% l-Glut (D2) and added to the wells at the specified MOIs. Following a 1 h absorption period, the viral inoculum was removed, the cells were washed twice with PBS and maintained in D2 media until sample collection. Samples were collected at designated timepoints when viral titres reached detectable levels (24 hpi for RVFV and 48 hpi for ZIKV) unless otherwise noted.

### Competitive inhibition studies

Cells were seeded as described above 1 day prior to infections, and cell lines used ranged from passage 4 to 18 depending on the experiment. For the mRAP studies, on the day of infection, cells were pre-treated with 5 µg ml^−1^ of mRAP_D3_, mutant mRAP_D3_, or D2 media as a control for 1 h at 37 °C. Viral inoculum was added to the wells (MOI 0.1), incubated for 1 h at 37 °C, washed twice with PBS and maintained in 2.5 µg ml^−1^ of mRAP_D3_, mutant mRAP_D3_, or D2 media control. The cells were then incubated at 37 °C overnight, and supernatant samples or coverslips were collected at 24 hpi. Samples were analysed at 24 hpi since mRAP_D3_ impacts early viral entry processes and is subsequently bound and cleared by LRP1 over time. For studies utilizing CL_II_ and CL_IV_ as decoys, RVFV ZH501 was diluted to an MOI 0.1 in DMEM containing 1% Pen/Strep and 1% l-Glut, and then the viral inoculum was pre-treated with 5 µg ml^−1^ each LRP1 CL_II_ and CL_IV_ proteins (Biotechne, 2368-L2-050 and 5395-L4B-050) or no treatment control in the same media for 1 h at 37 °C with mixing every 15 min. Following pre-treatment, the viral inoculum containing decoys was used to infect the cells as described above.

### Immunofluorescent imaging

For imaging, 2.5E5 cells were seeded on l-lysine-coated coverslip the day prior to infection. At the time of harvest, the cells were fixed with 4% paraformaldehyde, permeabilized with 1% Triton X-100 for 15 min at room temperature, washed with PBS and then blocked with 5% normal goat serum for 1 h at room temperature. Following blocking, the cells were then washed again with PBS, and the primary antibody solution was added (diluted in 5% normal goat serum) and incubated for 1 h at room temperature. Following the primary antibody incubation, the cells were again washed, and the secondary antibodies were added (diluted in 5% normal goat serum) for 1 h at room temperature. Lastly, the cells were washed and Hoechst stain was added for 30 s. The Hoechst stain was removed, and the cells were washed and mounted on glass slides with gelvatol.

### Viral quantification by VPA and qPCR

Supernatant samples from RVFV-infected cells were collected at the indicated timepoints. vRNA was quantified via quantitative reverse transcription polymerase chain reaction (qRT-PCR) and infectious titres were analysed via viral plaque assay from the cell supernatant as previously described [34]. We quantified vRNA in unknown samples by comparing their cycle threshold (Ct) values to a standard curve generated in-house from known RVFV ZH501 RNA standards. To create this standard curve, we prepared serial dilutions of virus at known concentrations (measured in p.f.u.) and determined their corresponding Ct values. We then used this established curve based on Ct values to extrapolate viral titres in our unknown samples, expressing the results as p.f.u. equivalents per mL. ZIKV vRNA was measured as described above with the following primers: 5′-CTGTGGCATGAACCCAATAG-3′ and 5′-ATCCCATAGAGCACCACTCC-3′ and probe: 5′-CCTTTGCAGCTGGAGCGTGG-3′ labelled at the 5′ end with the reporter 6-FAM and internally quenched with a modified ‘T’ residue with BHQ1 and a modified 3′ end to prevent probe extension. The LOD for vRNA in the qRT-PCR reaction is 2,560 p.f.u. equivalents ml^−1^ for RVFV and 17 p.f.u. equivalents ml^−1^ for ZIKV.

### Protein and antibody reagents

WT mRAP_D3_ and mutant mRAP_D3_ (K265A/K279E) were generated as previously described [2, 4, 6]. Recombinant LRP1 Cluster II–Fc (CL_II_) and LRP1 Cluster IV–Fc (CL_IV_) were purchased from R&D Systems (2368-L2 and 5395-L4B, respectively). The following antibodies were used for immunofluorescence staining: mouse anti-RVFV NP (1:100, BEI, 43199) and rabbit anti-LRP1 (1:200, Abcam, ab92544), and secondaries goat anti-rabbit 488 (1:500; Invitrogen, A11008) and goat anti-mouse 594 (1:500; Invitrogen, A11012). The following antibodies were used for Western blots: rabbit anti-LRP1 and mouse anti-beta-actin (1:500, Santa Cruz Biotechnology, Sc-47778), and secondaries goat anti-rabbit IRDye 800CW (1:10,000; LiCORbio 926–32211) and goat anti-mouse IRDye 680RD (1:10,000; LiCORbio 926–68070).

### Western blot

Protein lysates were generated from JEG-3, JAR and HTR-8 cells in radioimmunoprecipitation assay buffer (Thermo Fisher Scientific, 89901) with 1% Halt Protease Inhibitor (Thermo Fisher Scientific, 78429). Since subsequent experiments were conducted on various passages of each cell line (ranging from passage 4 to 18), protein lysates from cells at various passages were harvested and analysed for LRP1 expression. The cell protein lysates were centrifuged to remove cellular debris, and the protein concentration was quantified using a bicinchoninic acid assay (Thermo Scientific, 23225). 30 µg of protein from each sample was loaded on a NuPAGE 4–12% Bis-Tris gel (Invitrogen, NP0323BOX) and ran for 30 min at 165 V. The proteins were then transferred to a LICOR membrane using the iBlot 2 system (pre-set programme P0, Invitrogen, IB21001). The membrane was blocked with Intercept (PBS) Blocking Buffer (LI-COR, 927–70001) and then probed with primary (see reagent section above for specific information) antibodies diluted in Intercept T20 (PBS) Antibody Diluent (LI-COR, 927–75001) rocking at 4 °C overnight. The membrane was washed three times with PBS 0.1% Tween (PBS-T), and then the membranes were probed with secondary antibodies for 1 h at room temperature. The membrane was washed three times in PBS-T and then stored in PBS until visualization. The membrane was visualized utilizing an Odyssey CLx Imaging system (LI-COR) and analysed with Image Studio (LI-COR) software. LRP1 expression was normalized to beta-actin and expressed as signal density.

### Statistical analyses

GraphPad version 9 (Prism) was used for all statistical analyses. Two-way ANOVAs and unpaired two-sample t-tests were utilized to measure significance where noted.

## Supplementary material

10.1099/jgv.0.002300 Supplementary Material 1.

## Abbreviations

ATCC: American Type Culture Collection
CDC: Centers for Disease Control and Prevention
CLII: cluster II
CLIV: cluster IV
Ct: cycle threshold
DMEM: Dulbecco’s Modified Eagles Medium
EMEM: Eagle’s Minimum Essential Medium
FBS: fetal bovine sera
hpi: hours post-infection
JCV: Jamestown Canyon virus
LDL: low-density lipoprotein
LDLR: low-density lipoprotein receptor
LOD: limit of detection
LRP1: lipoprotein receptor-related protein 1
MOI: multiplicities of infection
mRAPD3: Murine RAP domain 3
no Tx: no treatment
OROV: Oropouche virus
p.f.u.: plaque-forming units
qRT-PCR: quantitative reverse transcription polymerase chain reaction
RAP: receptor-associated protein
RBL: Regional biocontainment laboratory
RPMI: Roswell Park Memorial Institute 1640 medium
RVFV: Rift Valley Fever virus
vRNA: viral RNA
ZIKV: Zika virus
