# Supplementary material for: Rift Valley fever virus infects trophoblast cell lines in an LRP1-dependent manner
Source: J Gen Virol. 2026 Aug 12;107(8):002300. doi: 10.1099/jgv.0.002300 (PMC13465766; doi:10.1099/jgv.0.002300)
Supplement: Supplementary file 1 [file jgv-107-02300-s001.pdf]

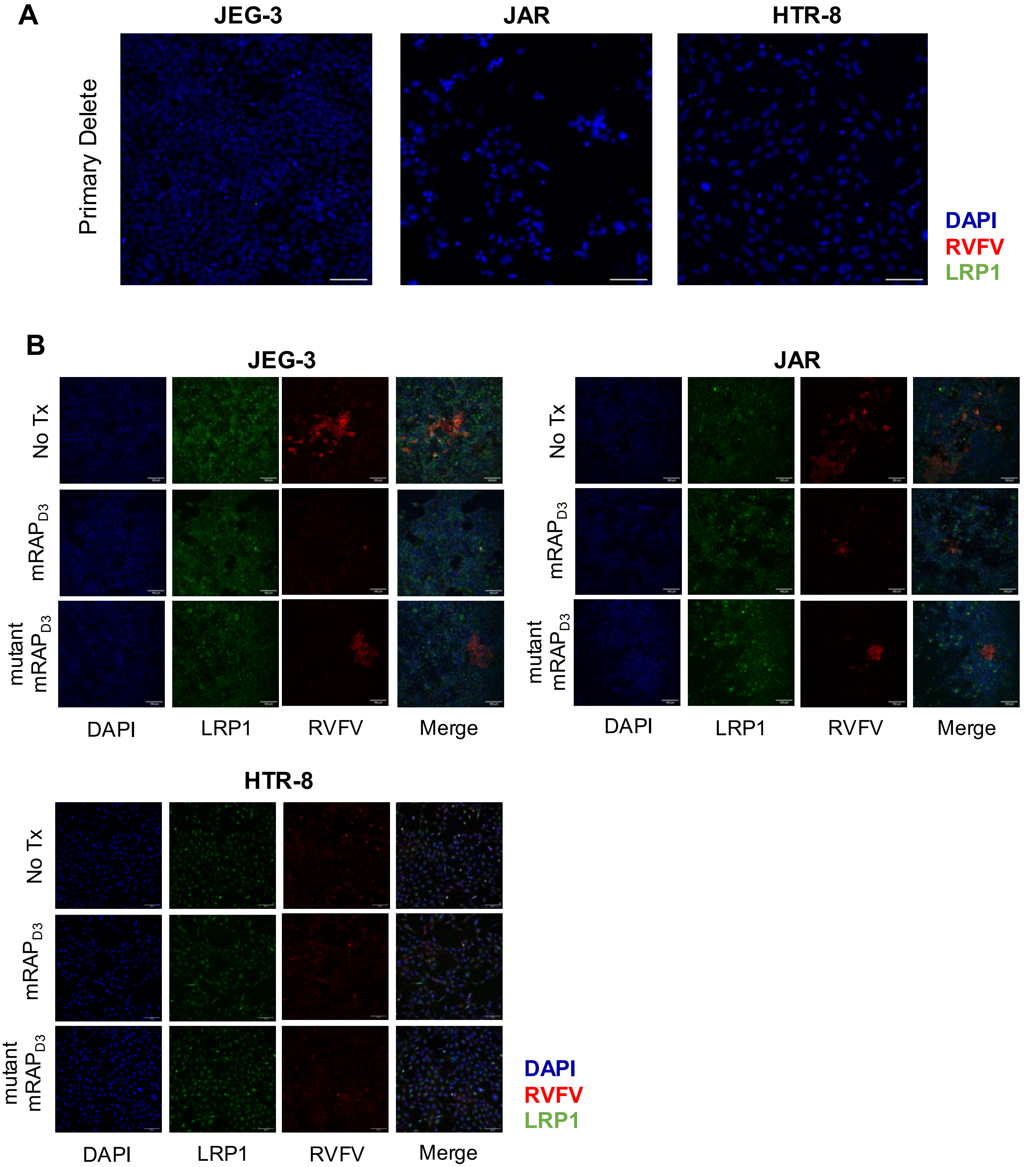

Supplemental Figure 1: Immunofluorescent Images. (A) Primary delete control images from immunofluorescence staining experiments in Figure 1 and 2. (B**)** Individual channel images demonstrating wildtype mRAP$_{D3}$ treatment (5 µg/mL) reduced RVFV (MOI 0.1) titers in JEG-3, JAR and HTR-8 cells. images obtained at 20x, scale bar = 100 µm. images obtained at 20x, scale bar = 100 µM.
